# Optimized imaging of the midface and orbits

**DOI:** 10.3205/cto000120

**Published:** 2015-12-22

**Authors:** Sönke Langner

**Affiliations:** 1 Institute for Diagnostic Radiology and Neuroradiology, University Medicine Greifswald, Germany

**Keywords:** imaging, midface, orbits, CT, MRI

## Abstract

A variety of imaging techniques are available for imaging the midface and orbits. This review article describes the different imaging techniques based on the recent literature and discusses their impact on clinical routine imaging. Imaging protocols are presented for different diseases and the different imaging modalities.

## 1 Introduction

The complex anatomy of the nose, the paranasal sinuses, the orbits, and the adjacent anatomical compartments of the neck poses a diagnostic challenge for both clinical examinations and diagnostic imaging.

Traumatic lesions usually present with unique clinical features, and high-resolution imaging is needed to precisely describe their anatomical location and extent [1], [2]. On the other hand, clinical signs of tumors of the midface are often unspecific [3], [4]. Therefore, most patients have advanced disease at the time of diagnosis [4]. In tumor patients, imaging is needed to precisely evaluate the local tumor extent, especially if the area is not accessible clinically, and to identify lymphatic involvement and distant metastases. In oncologic patients, imaging is needed to monitor the treatment response and to differentiate between residual and recurrent tumor or treatment-related changes [4].

This review will present the different imaging modalities for sinonasal and orbital imaging, and their clinical relevance will be discussed with respect to the recent literature. Imaging protocols for the different modalities and disease entities will be provided.

## 2 Conventional radiography (CR)

Conventional radiography is a projection technique generating a two-dimensional (2D) image. The increasing availability of flat panel detectors for image detection has significantly decreased radiation exposure [5], because flat panel detectors are more sensitive to radiation and provide better image quality with less radiation exposure [6]. 

**Figure 1:**
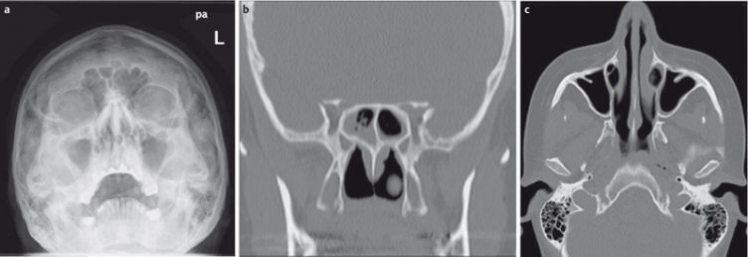
53-year-old patient with leukemia prior to radiation therapy. a) Conventional radiograph of the paranasal sinuses; soft tissue swelling within the right maxillary sinus without air-fluid level. b) CT of the paranasal sinuses of the same patient. The CT was performed 2 hours after plain radiographs. CT shows sinusitis of the sphenoid sinus not detectable on the radiographs. c) CT does not confirm sinusitis of the right maxillary sinus.

Because the different structures are projected onto each other, conventional radiographs (CR) are of limited diagnostic value (Figure 1 (Fig. 1)) for assessment of the paranasal sinuses and have become obsolete in the diagnosis of acute or chronic rhinosinusitis. They are therefore not recommended by the major societies [7], [8]. In a prospective study of 134 patients who underwent CR and computed tomography (CT) examinations of the paranasal sinuses on the same day, Konen et al. found CR to be inferior to CT in diagnostic accuracy [9]. The sensitivity of CR for the detection of inflammatory changes of the maxillary sinus was 67.7%, but only 14.6% and 3.8% for the ethmoid and sphenoid sinus, respectively, compared to low-dose CT (ldCT) (Figure 1 (Fig. 1)). 

Because soft tissue changes are only poorly visualized, CR plays no role in tumor imaging [8].

**Figure 2:**
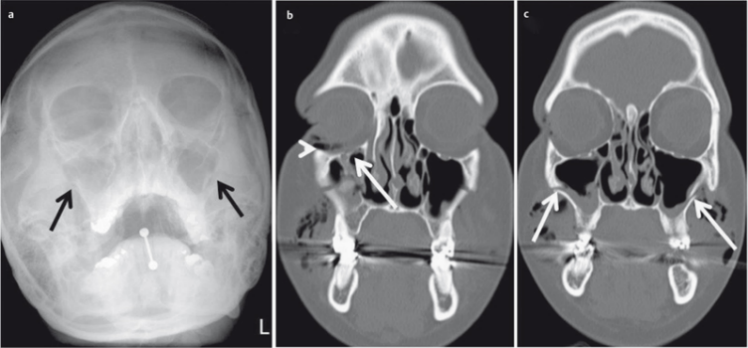
22-year-old drunken patient having received blows to the face. a) Conventional radiography of the paranasal sinuses. Suboptimal image quality due to lack of compliance. There is a fracture of the lateral aspects of both maxillary sinuses (arrow), additional fractures are not detectable. b) CT of the midface, coronal plane; fracture of the floor of the orbit (arrow) and intraorbital air (arrowhead). c) CT of the midface allows precise determination of the extent of the fracture (arrow).

Kim et al., in a retrospective study of 92 patients, found a sensitivity of 85.7% and 88.7% sensitivity for the detection of fractures of the orbital floor or the lamina papyracea in comparison to CT [10]. The authors claim that fractures that were missed by CR did not need surgical treatment; however, they do not provide any details on functional outcome in the patients with missed fractures on CR. Furthermore, herniation of orbital content cannot be appreciated accurately on CR. Therefore, a CT should be obtained when a facial fracture is suspected clinically (Figure 2 (Fig. 2)) [1], [8], [11], [12]. 

## 3 Digital volume tomography (DVT)

**Figure 3:**
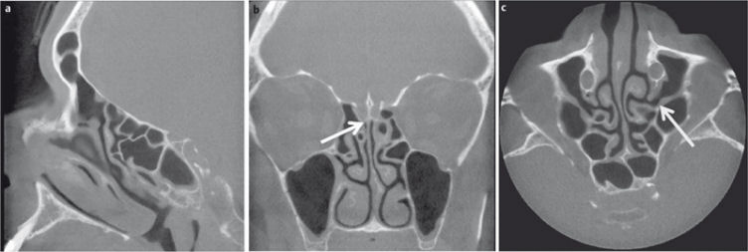
DVT of a 43-year-old patient with clinically suspected exacerbation of chronic rhinosinusitis. a) Sagittal plane at the level of the fronto-ethmoid recess (arrow). b) Coronal plane at the level of the cribriform plate (arrow). c) Axial plane at the level of the ostiomeatal complex (arrow).

Digital volume tomography (DVT) or “cone beam tomography” is a recent technical development based on the principle of orthopantomography. As with CT, the X-ray beam in DVT rotates around the patient but is conical in configuration, while it is fan-shaped in CT [13]. During a rotation, which may cover 180° or 360°, multiple 2D X-ray images are recorded, which are then assembled into a three-dimensional (3D) volume dataset by the application software [13]. This dataset can then be reviewed in any plane (Figure 3 (Fig. 3)) or be further processed three-dimensionally.

DVT systems for clinical use have been available since 1982. With their wider availability, they are increasingly being used for different ear, nose, and throat (ENT) applications [8], [14]. 

**Figure 4:**
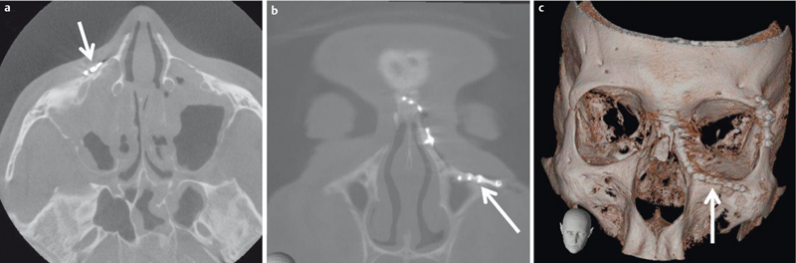
DVT of 19-year-old patient after surgery for a complex fracture of the midface. a) Axial plane; only discrete artifacts caused by the osteosynthetic material (arrow). b) Coronal plane demonstrating the position of the small plate (arrow). c) 3D VRT reconstruction (volume rendering technique, VRT) confirming correct placement of the implants.

DVT systems produce images with a highly isotropic (i.e., the dimension of the volume pixel, or voxel, is the same in all three spatial directions) local resolution at relatively low radiation exposure [14], [15], [16]. With the radiation dose used and the postprocessing algorithms available, DVT allows the delineation of fine bony structures [17] and is less sensitive to artifacts of metal dental implants (Figure 4 (Fig. 4)) than CT [18]. Various commercial DVT systems are available for clinical routine imaging with the patient in different positions (standing, sitting, or lying). Unlike CT, the volume of interest (VOI) has to be defined in DVT prior to image acquisition. This VOI depends on the indication for imaging [18] and should include a cylinder of 10×10 cm when imaging the paranasal sinuses. For imaging the nasal skeleton, a cylinder of 4×4 cm is considered sufficient. 

With its excellent spatial resolution, DVT is considered a valid alternative to CT in the diagnosis of fractures. Because DVT systems were initially used for imaging of dental fractures [14], comprehensive scientific data are available. However, there is also an increasing number of publications on the use of DVT in imaging facial fractures [19]. In a prospective patient population, Choudhary et al. [20] demonstrated the superiority of DVT over conventional radiography for the detection of midfacial fractures. However, in the acute trauma setting, DVT is rarely used because positioning of the patient in the DVT system is not possible and CT continues to be the preferred imaging modality [17], [21]. 

**Figure 5:**
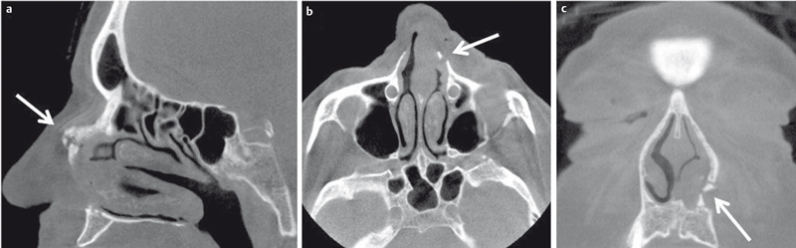
83-year-old female patient who fell due to cardiac event. DVT allows identification of the precise anatomical location of the fragment of the nasal fracture in all planes (a = sagittal, b = axial, c = coronal).

**Figure 6:**
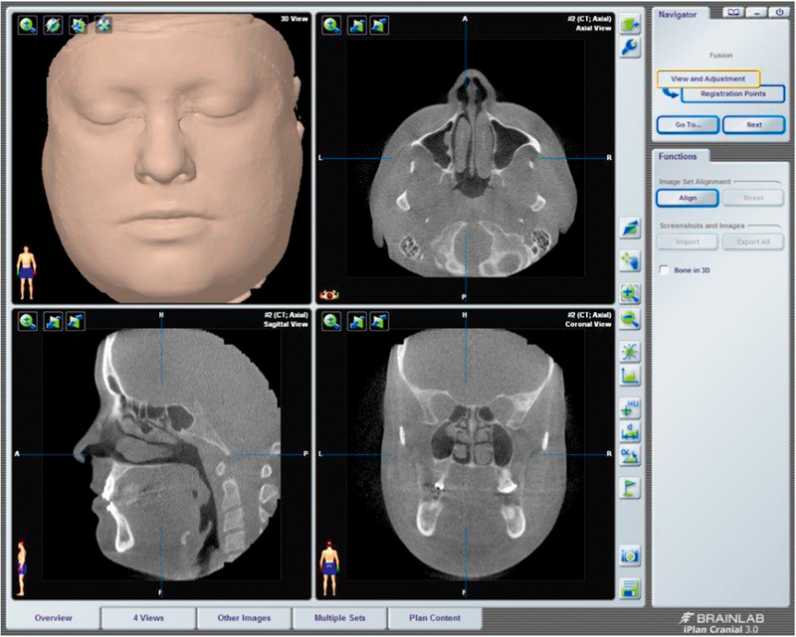
Neuronavigation (brainlab^©^) using a DVT dataset.

In a prospective collective of 65 patients, Bremke et al. found DVT to be superior to CR in the detection of nasal fractures [19]. While only 5 fractures were missed on CR, exact spatial mapping of the fracture and of the dislocated fragments was improved using DVT (Figure 5 (Fig. 5)). DVT is also suitable for postoperative imaging after osteosynthetic treatment of facial fractures because it is less sensitive to metal artifacts than CT (Figure 4 (Fig. 4)). DVT datasets can also be used for neuronavigation with some clinical neuronavigation systems. However, in these cases, a large VOI is required (Figure 6 (Fig. 6)).

**Figure 7:**
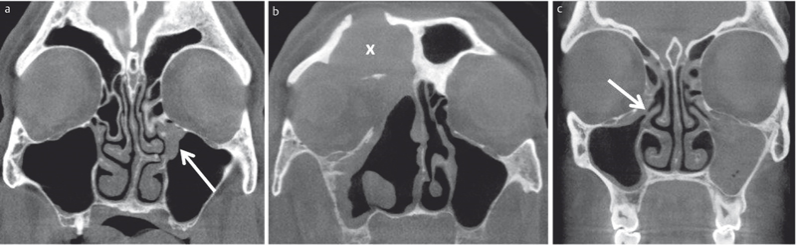
DVT images in coronal plane in different patients with inflammatory changes of the paranasal sinuses. a) 56-year-old female patient with chronic rhinosinusitis presenting with new onset of pain and feeling of pressure in the left maxillary sinus. Obstruction of the ostiomeatal complex (arrow). b) 72-year-old patient with chronic sinusitis and multiple surgical treatments. Exacerbation and new onset of pain in the right maxillary sinus. DVT demonstrates inflammatory tissue swelling. The mucocele of the frontal sinus was asymptomatic. c) 22-year-old female patient with recurrent rhinosinusitis and bilateral facial pain. Complete opacification of the left maxillary sinus and obstruction of right ostiomeatal complex.

Another important application of DVT is imaging of the paranasal sinuses (Figure 7 (Fig. 7)) in patients with chronic rhinosinusitis [14], [16], [18], [22]. Leiva-Salinas et al. [15] prospectively evaluated 40 patients with inflammatory changes of the paranasal sinuses who underwent paranasal DVT and CT within 1 hour. Although image quality of DVT was inferior to that of CT, this did not affect diagnostic accuracy while significantly decreasing radiation exposure. This is of particular importance because the majority of patients with rhinosinusitis are young adults [15], in whom reduction of radiation exposure is a crucial concern [23], [24], especially if repeated imaging is needed in chronic disease [25]. In these cases, magnetic resonance imaging (MRI) has to be considered as an alternative imaging modality [8].

Radiation exposure is higher for a conventional CT scan of the paranasal sinuses compared to a DVT study [15]. However, DVT systems have a wide range of effective doses applied to the patient as demonstrated in a study by Rottke et al. [16], who evaluated 10 different DVT systems. These results were reproduced by other authors. Therefore, these investigators recommend optimization and critical review of the default DVT study protocols provided by manufacturers [15], [16], [26]. 

**Figure 8:**
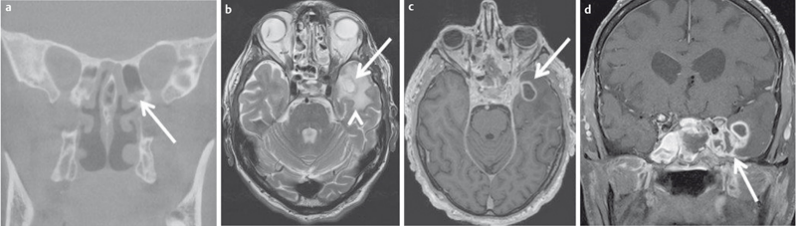
67-year-old diabetic patient with numb centrally located headache. a) Coronal reconstructed DVT demonstrates inflammatory changes of the posterior ethmoid cells (arrow) and the sphenoid sinus on the left side. b) New onset of seizure a few hours after the DVT. T2w MR images demonstrate a ring-like mass (arrow) with surrounding edema (arrowhead). c) The lesion displays homogenous ring-like enhancement on axial T1w images. d) Contrast-enhanced coronal T1w images demonstrate extension to the dura and the thrombosed cavernous sinus. Fungal sinusitis was confirmed by surgery.

For technical reasons, DVT provides inferior soft tissue contrast. Therefore, DVT plays only a minor role in the diagnosis of facial tumors and is inferior to CT and MRI in detecting complications of sinusitis (Figure 8 (Fig. 8)). Fakhran et al. [22] analyzed how many soft tissue processes are missed by DVT examinations. In their retrospective study of 361 patients included over a period of one year, they generated virtual DVT datasets from conventional CT examinations of the midface. The authors rated any soft tissue process that was not located on a border with an air-filled structure as not detected by DVT. They were able to show that, with this approach, less than 3.5% of patients had a lesion that would have been missed by DVT. Additionally, these potentially missed lesions were already known in 2/3 of the patients. These results indicate that appropriate patient selection is of high clinical relevance. In immunocompromised patients or in patients with suspected invasive fungal sinusitis or with orbital or intracranial complications, CT should be performed [22].

The use of contrast agents in DVT examinations to improve soft tissue contrast is still experimental [27]. Alsufyani et al. showed that the mucosal surface of the paranasal sinuses is best opacified by using 25% barium sulfate, while iodine-containing contrast agents are not suitable [28]. However, even with this approach, the authors failed to reliably opacify soft tissues cranial to the middle turbinate [28].

Studies comparing DVT and CT are still limited. The current guidelines list DVT as a possible alternative for head and neck imaging [7], [21], [29] but make no specific recommendations [8].

## 4 Computed tomography (CT)

Computed tomography (CT) is the most commonly used modality for head and neck imaging [7], [8]. Major advantages of CT are the availability and the short examination time, which is well tolerated by the majority of patients. Another, increasingly more important aspect are the costs in comparison to MRI [30].

For a CT examination, the patient lies on a table with the tube rotating around the patient. In contrast to DVT, the X-ray beam is fan-shaped. Opposite to the tube is the detector, which registers beam attenuation and converts it into a grayscale image. The grayscale of each voxel can be quantified in Hounsfield units, which are used to characterize different tissues [31]. 

**Figure 9:**
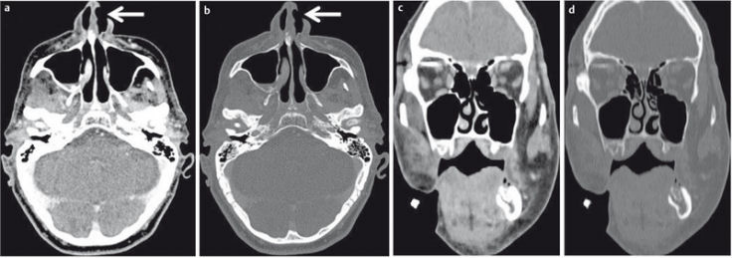
67-year-old patient after surgical treatment of a malignant melanoma of the left nose. CT of the midface in axial (a,b) and coronal (c,d) planes with different window-level settings (soft tissue window level: a,c; bone window level: b,d). The window-level setting has to be chosen according to the tissue to be evaluated.

The different grayscale values which can be differentiated by the eye have to be allocated to a specific range of Hounsfield units, the so-called “window-level-setting”, to improve image contrast. Different window-level settings are available, from which the radiologist must choose the best one for a given clinical question to be answered (Figure 9 (Fig. 9)).

**Figure 10:**
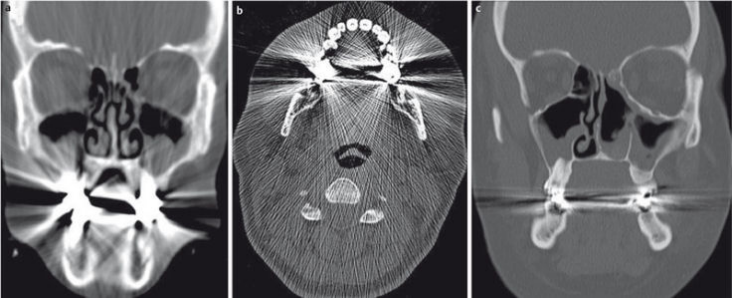
The occurrence of artifacts depends on the primary acquisition plane of the CT. a) 32-year-old female patient; CT of the paranasal sinuses acquired in primary coronal plane. Reduced image quality and diagnostic accuracy due to severe artifacts. b) 37-year-old patient with chronic rhinosinusits. CT acquired in primary axial plane is degraded by severe artifacts due to dental implants. c) Secondary reconstructed coronal plane of the same patient as in b. The artifacts are limited to the acquisition plane. Therefore, image quality and diagnostic accuracy of the paranasal sinuses are not reduced.

The multidetector CT (MDCT) scanners available in clinical routine yield high-resolution axial volume datasets with submillimeter spatial resolution [32]. Images in different planes can be reconstructed secondarily from these datasets without the need for additional radiation exposure or a loss of image information [33]. Primary coronary CT scans of the paranasal sinuses have become obsolete [34] because artifacts caused by metal dental implants are significantly enhanced (Figure 10 (Fig. 10)) compared to axial scans [34], [35]. Tumor imaging cannot be performed without IV administration of an iodine-based contrast agent. These contrast agents are hypo- or iso-osmolar to serum and have a low profile for adverse events [36].

### 4.1 Fractures of the midface

**Figure 11:**
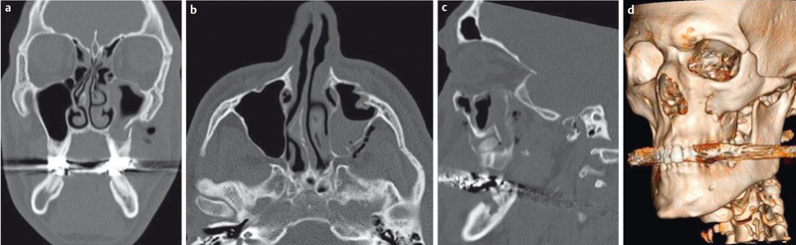
19-year-old patient who fell on the face. CT of the midface in bone window-level setting (a–c) demonstrates the exact extent of the complex fracture of the maxillary sinus, zygomatic arch (“tripod fracture”), and the floor of the orbit. 3D-VRT reconstruction demonstrates the anatomical relationship of the different fragments.

**Figure 12:**
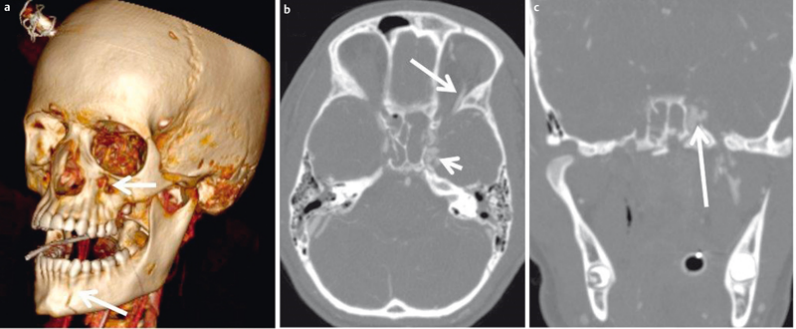
16-year-old patient with severe trauma to the head and progressive exophthalmos and chemosis. a) 3D-VRT reconstruction demonstrates the extent of the fracture with only minimal displacement of the fragments. b) Contrast-enhanced CT angiography demonstrates opacification of the cavernous sinus (short arrow) and the superior ophthalmic vein (long arrow) indicating carotid cavernous sinus fistula. c) Coronal MIP reconstruction (maximum intensity projection, MIP) demonstrates the fistula (arrow).

Midface injuries occur in approximately 25% of all trauma patients [37]. In addition to the clinical examination, imaging is crucial for the management of these patients. The imaging modality of choice is CT [38] because the midfacial CT scan is usually part of the routine trauma CT scan or can be performed immediately afterwards [39]. Planning of surgery is often facilitated by 3D preoperative planning (Figure 11 (Fig. 11), Figure 12 (Fig. 12)) [37]. 

### 4.2 Imaging of paranasal sinuses

Sinusitis is the most common disease of the paranasal sinuses and has both a clinical and a socioeconomic impact. Acute sinusitis is a clinical diagnosis and imaging is not required routinely. In chronic sinusitis, however, imaging is needed for treatment planning.

**Figure 13:**
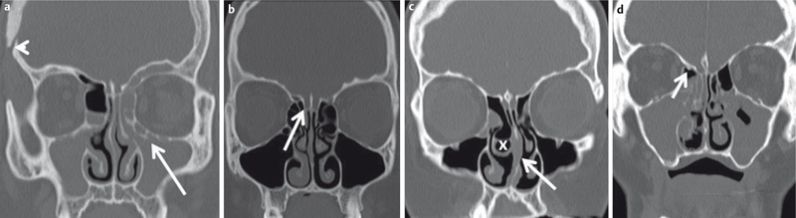
Relevant anatomical variants of the paranasal sinuses prior to FESS. a) 43-year-old patient with chronic rhinosinusitis and recurrent surgery; complete opacification of the maxillary sinus on both sides. Haller cell (arrow) on the left side causing obstruction of the left ostiomeatal complex along with an ethmoid bulla. The patient underwent cranial surgery (arrowhead) due to meningioma. b) 19-year-old female patient; ethmoid bulla on the left side. Keros type II Cribriform plate variant. c) 37-year-old patient prior to surgery. Concha bullosa of the middle turbinate on the right side (x). Left convex deviation of the nasal septum (arrow) with small bony spur. Small Haller cell and opacified ethmoid bulla on the right side. d) 56-year-old patient with exacerbation of chronic rhinosinusitis after repeated surgery. Small bony spur in the course of the anterior ethmoid artery (arrow). Keros type I cribriform plate variant.

When imaging high-contrast structures such as the paranasal sinuses, higher image noise is acceptable. Therefore radiation exposure can be reduced [40] without a relevant loss of image quality or decrease in diagnostic accuracy. With the use of these so-called low-dose CT (ldCT) protocols it is possible to image the paranasal sinuses with radiation exposure similar to conventional radiographs and DVT [3], [25]. Therefore, ldCT is the imaging technique of choice in the diagnostic work-up of chronic sinusitis [7], [8], [18]. Compared to DVT, ldCT also provides an excellent display of the anatomy and the anatomical variants of the paranasal sinuses (Figure 13 (Fig. 13)) before Functional Endoscopic Sinus Surgery (FESS) [41]. In a phantom study, Kröpil et al. [42] demonstrated that radiation exposure of ldCT can be further reduced by the use of special post-processing filters. However, this technique needs processing steps. In another phantom study, Schulz et al. demonstrated that similar effects can be achieved by using a special image reconstruction technique, so-called iterative reconstruction [43]. These experimental findings were confirmed by Bulla et al. in a feasibility study of 80 patients [44]. By using iterative methods of reconstruction they could reduce the dose of an ldCT protocol by 68% compared to a standard CT protocol. In contrast to DVT, the dose reductions that can be achieved are similar for different CT scanners. Therefore, CT examinations have a significantly lower variation in radiation exposure compared to DVT, as also demonstrated in a prospective study of Hoxworth et al. [45]. 

**Figure 14:**
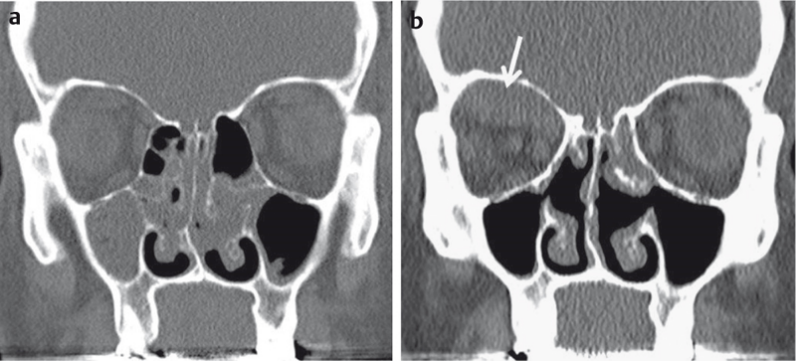
63-year-old diabetic patient with exacerbation of chronic rhinosinusitis. a) CT of the paranasal sinuses, coronal reconstructed image in bone window-level setting. Complete opacification of the right maxillary sinus and obstructed left ostiomeatal complex. b) Follow-up CT two weeks later after conservative therapy. New onset of headache and diplopia. Coronal reconstructed image in bone window-level setting demonstrates a smoothly marginated subperiostal lesion. Abscess formation was confirmed by surgery.

**Figure 15:**
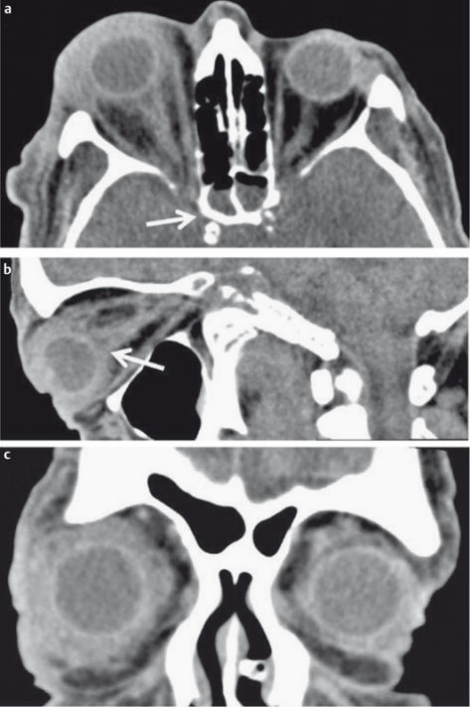
74-year-old diabetic patient with acute onset of nerve palsy of the 3^rd^, 4^th^ and 6^th^ cranial nerves on the right side and progressive “reddening” of the right eye and painful eye movement. a) Axial CT demonstrates inflammatory changes of the right sphenoid sinus (arrow). b) Sagittal reconstructed CT images demonstrate diffuse inflammatory changes of the globe and the optic nerve (arrow). c) Coronal reconstructed CT images demonstrate subtle inflammatory changes also on the left side. MRI (not shown) demonstrated thrombosis of the cavernous sinus on both sides, which was not detectable on CT. Surgery confirmed Candida infection.

CT is also the imaging technique of choice when complications of sinusitis are suspected (Figure 14 (Fig. 14)). However, in these patients, standard CT protocols should be used instead of ldCT and administration of contrast agent is mandatory [8], [46], [47]. However, CT is inferior to MRI for the detection of intracranial complications (Figure 15 (Fig. 15)) [46], [47]. 

**Figure 16:**
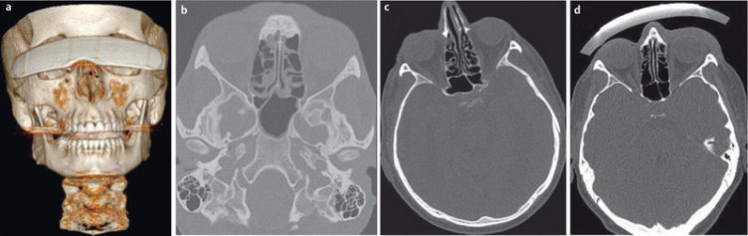
Impact of lens protection devices on image quality. a) 3D-VRT reconstruction. The lens protection device can be worn like glasses. b) Standard CT protocol of the midface without lens protection. c) Low-dose CT protocol without lens protection. Discrete increase of image noise. d) Low-dose CT protocol with lens protection. There is no significant decrease of image quality. Lens exposure is reduced by 58%.

**Figure 17:**
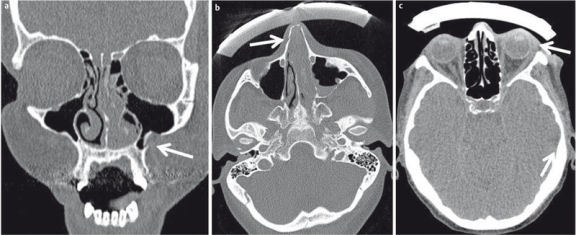
Low-dose CT of the midface using a 64-row dual-source CT scanner. a) 18-year-old female patient who fell on her face. Coronal reconstructed image with 3 mm slice thickness. The fracture (arrow) can be assessed precisely. b) 23-year-old patient with direct trauma to the head. Despite the lens protection device the fracture of the nasal bone is detected. c) 47-year-old patient with facial swelling on the left side. The preseptal cellulitis (arrow) is detected despite the lens protection device.

When imaging the midface, the eye lens – the most radiation-sensitive part of the body – lies within the beam [48]. The threshold for cataract induction specified in the literature is 0.5–2 Sievert (Sv) [49]. For fractional repeated radiation exposure, a threshold of 0.1 SV has been reported [50]. In a population-based retrospective evaluation of 2,276 patient and 27,761 controls, Yuan et al. calculated a *hazard ratio* of up to 2.12 and a percentage increase of up to 1.45% for cataract induction after repeated CT studies of the head and neck [51]. Although this is the study with the largest number of patients, its results are discussed controversially. One of the main criticisms is that the patient group consisted of cancer patients and the impact of concomitant chemotherapy was not taken into account [52]. A reduction of lens radiation can, in addition to modification of the scanning protocol or image reconstruction, further be achieved by a special lens cap (Figure 16 (Fig. 16)), which is worn like a pair of glasses [53]. In a phantom study, Keil et al. calculated a dose reduction for the lens with the use of such lens protection devices that ranged from 38–48%, depending on the material used [54]. The latest-generation CT scanners offer organ-related tube current modulation (TCM) [55], [56], [57]. Wang et al. showed, in a phantom study, that the synergistic effect of TCM and lens protection results in a significant reduction of 47% of the lens dose [58]. Plessow et al. [59] validated these results in a prospective study of patients examined on a latest-generation CT scanner. Their results show that lens protection devices and ldCT protocols can be used without a loss of diagnostic accuracy (Figure 17 (Fig. 17)).

### 4.3 Tumors of the midface

**Figure 18:**
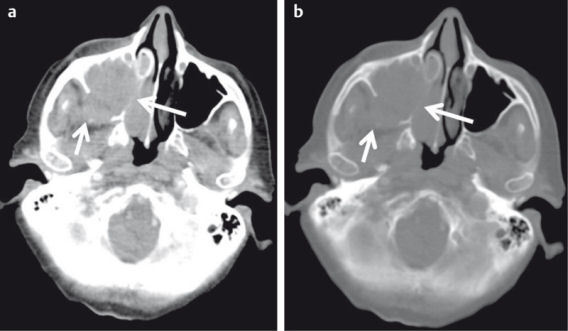
Contrast-enhanced CT of the midface with squamous cell carcinoma of the right maxillary sinus. a) Axial image in bone window level setting. Complete opacification of the right maxillary sinus with tumor extension to the retromaxillary fossa (short arrow) and the nasal cavity. Infiltration of the medial wall of the maxillary sinus. b) Infiltration of small bony structures can better be appreciated in bone window-level settings.

Tumors of the midface are rare and account for approximately 3% of all head and neck malignancies [60], [61]. Men are more often affected, and the peak age is between 50 and 70 years. CT is the imaging method of choice due to its availability and the short examination times. However, MRI is superior for the detection of submucosal or perineural tumor spread (see MRI section). In comparison CT is superior to MRI for the detection if osseous infiltration of small bony structures is suspected (Figure 18 (Fig. 18)). Therefore both imaging modalities are often considered as complementary in head and neck imaging [4], [30]. For diagnostic workup in tumor patients, CT protocols with higher tube currents (>50 mAs) should be used, and IV administration of an iodine-based contrast agent is always required [30]. To minimize the artifacts by metallic dental implants, primary axial thin-layer datasets with secondary multiplanar reconstruction (MPR) in bone and soft tissue window-level settings should be acquired [4].

**Figure 19:**
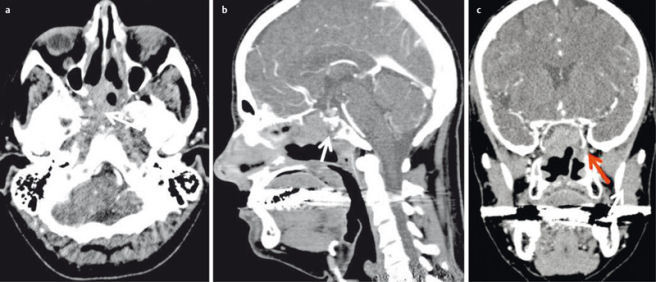
Contrast-enhanced CT of the midface of a 47-year-old patient after resection of a squamous cell carcinoma of the nasal cavity and new onset of epistaxis. a) Axial plane; recurrent disease with posterolateral infiltration of the sphenoid sinus and extension into the carotid canal (arrow). b) Sagittal plane demonstrating osseous infiltration of the clivus (arrow). c) Coronal plane with extension of the tumor into the pterygopalatine fossa (arrow).

The main task of imaging is to exactly describe the local tumor extent (Figure 19 (Fig. 19)) and to detect possible lymph node and distant metastases [4], [30], [62].

Conventional CT imaging with static contrast agent administration mainly identifies morphological changes and provides anatomical information [63]. No conclusions regarding tumor physiology or biological behavior can be drawn. Perfusion CT (PCT) is a so-called functional imaging technique and allows noninvasive assessment of vascular architecture and the blood flow status of a tumor. It therefore offers quantifiable biomarkers [64]. 

**Figure 20:**
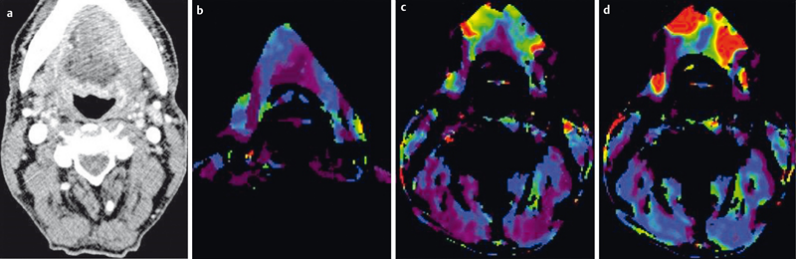
Perfusion-CT of a 51-year-old patient with lip carcinoma and newly diagnosed induration of the anterior floor of the mouth. a) Axial contrast-enhanced CT demonstrates discrete asymmetry and mild contrast enhancement on the left side. b) PCT demonstrates increased blood volume, c) increased blood flow d) and increased permeability surface product indicating malignancy. Metastasis was confirmed by histology.

Perfusion CT (PCT) relies on the sequential acquisition of CT images at a specific position or within a specific volume during the first passage of a bolus of contrast agent through the tissue. Contrast media induce a temporary increase in tissue density, and a time-density curve (TDC) can be calculated for each voxel. Mathematical models are used to calculate several perfusion parameters, including blood flow (BF), blood volume (BV), mean transit time (MTT), and the so-called *permeability-surface area product* (PS) (Figure 20 (Fig. 20)). The PS (in ml/min/100 g tissue) is a surrogate parameter for the presence of immature, neoangiogenetic vessels [64]. MTT (in sec) describes the average time the contrast agent needs for the first pass through the tissue. BV (in ml/100 g tissue) is a marker of the vascularity of the tumor, and BF (in ml/min/100 g tissue) allows quantification of blood flow within tumor [65].

In a retrospective study of 25 patients with advanced tumors, Hoefling et al. showed that there is a significant correlation between the PCT parameters and other relevant prognostic biomarkers [66]. Patients with lymph node metastases have a significantly poorer prognosis. However, identification of metastatic involvement of lymph nodes still relies on morphological criteria and is particularly difficult for lymph nodes <1 cm in diameter [67]. Bisdas et al. [68] and Trojanowska et al. [69] demonstrated that BF and BV are elevated in metastatic lymph nodes, though the difference to non-metastatic lymph nodes was statistically not significant in the study of Bisdas et al. Furthermore, PCT allows prognostically relevant characterization of the tumor with respect to its expected behavior during radiochemotherapy. Several studies suggest that there is a good correlation between increased BV and the response to radiochemotherapy [70], [71], [72], whereas low BF correlates with a poor response [73]. Another good correlation has been reported for the degree of malignancy and MTT [74]. Some authors recommend the use of PCT to monitor the effect of radio-chemotherapy [70], [71]. Since both postoperative changes and recurrent tumor display enhancement, differentiation between these two tissues can be difficult. In a retrospective study of Jin et al. [75], who investigated 48 patients with clinically suspected recurrence, an increase in BF identified recurrent tumor with 92.6% sensitivity and 96.3% specificity.

### 4.4 Orbit

Besides the detection of orbital complications of sinusitis (Figure 15 (Fig. 15), Figure 16 (Fig. 16)), CT is mainly used for orbital imaging in the acute trauma setting. Due to the superior soft tissue contrast, MRI has replaced CT in tumor patients [76], [77]. This also applies to the diagnosis of retinoblastoma [78]. 

**Figure 21:**
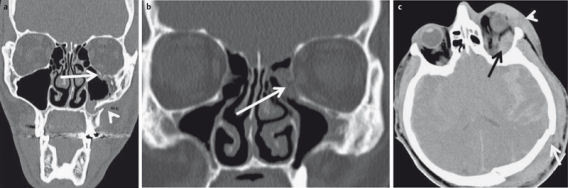
CT of the midface for detection of orbital fractures. a) 23-year-old football player with direct trauma to the left face and new onset of diplopia. Fracture of the floor of the orbita (arrow) with herniation of extraconal fat. b) 41-year-old patient with direct trauma to the globe. Fracture of the medial wall of the orbita and herniation of the medial rectus muscle (arrow). c) 86-year-old patient with severe head injury and rapid onset of exophthalmos. Intraorbital extraconal hematoma (black arrow) and periorbital hematoma (arrowhead). Impression fracture of the skull (white arrow).

The diagnostic work-up of orbital trauma also requires high-resolution axial planes in bone and soft tissue window-level settings with multiplanar reconstruction (Figure 21 (Fig. 21)) at least in axial and coronal planes [1], [11], [12]. It is very important to use thin-slice images with a decent overlap in order not to miss a fine fracture [33]. Particularly, the optic canal should be closely evaluated. In patients with complex fractures and roughly dislocated fragments, a 3D reconstruction can facilitate surgical treatment [12], [79]. IV administration of contrast agent is mandatory if inflammatory or infectious disease is suspected. Again, high-resolution thin slices in axial and coronal planes in soft tissue and bone window-level settings should be reconstructed with the latter being superior for the detection of osseous destruction. 

**Figure 22:**
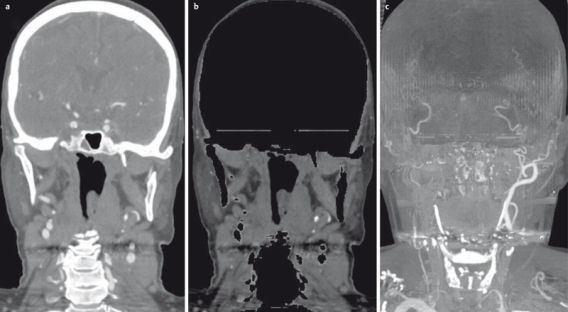
Dual-source CT of the midface. a) Fusion image of the 80 kV and 120 kV datasets. b) Automatic bone removal based on the different attenuation of bone in the two datasets. c) MIP reconstruction of the automatically segmented vessels.

A recent development in CT technology is the so-called dual-source CT (DSCT) scanner. With these CT scanners, the patient is examined by two different X-ray beams with different tube voltage. These beams can either be produced by separate X-ray tubes or by one tube with alternating tube voltages [80]. Because X-ray absorption within the tissue depends on tube voltage, two different datasets are generated, which can be further processed [81]. In addition to the automatic segmentation and removal of the bone (Figure 22 (Fig. 22)) for CT angiography, the DSCT scanner can be used for a variety of other applications in head-neck imaging, offering for instance advanced tissue characterization [80] or dose reduction protocols. By combining the two sets of data, overall image quality and lesion detection can be improved as demonstrated by Tawfik et al. in a prospective study of 60 patients [82]. Wichmann et al. presented a prospective study of 170 patients who underwent a DSCT. The dataset acquired with 80 kV was comparable to the standard protocol with 120 kVA in terms of diagnostic accuracy while resulting in a significant reduction of radiation exposure [83].

## 5 FDG-PET/Computed tomography (FDG-PET/CT)

**Figure 23:**
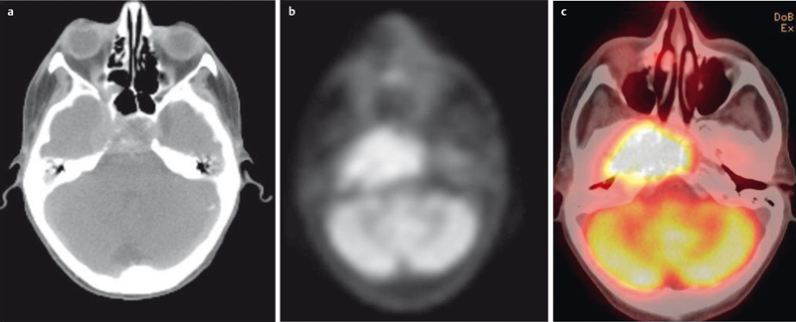
FDG-PET/CT is a hybrid imaging technique which fuses functional PET data with high-resolution CT datasets. a) High-resolution CT for computation of attenuation-corrected images and precise anatomical localization of pathological metabolism. b) Corresponding attenuation-corrected PET image. c) Fusion-CT for anatomical correlation.

Positron emission tomography (PET) is an imaging procedure that allows imaging of metabolism in vivo using a radioactively labeled tracer. While CT and MRI detect pathologies on the basis of morphologic changes, PET can demonstrate pathological metabolism in organs that appear morphologically inconspicuous [84]. PET with fluorine-18-fluoro-deoxy-D-glucose (FDG) is an established technique for diagnosis, staging, and follow-up of patients with tumors in the head and neck region [85], [86], [87], [88]. FDG is used as an analog for glucose in metabolism and is absorbed by the cells. Tumor cells usually have a higher metabolism than healthy cells, resulting in an increased uptake of FDG. Contrary to glucose, FDG is not further metabolized and therefore accumulates in tumor cells and can thus be detected. Usually, lesion characterization relies on the so-called SUV value (*standard uptake value*, SUV), which is a measure of the enrichment of the tracer within the lesion in relation to the total amount of tracer and the patient’s body weight [84]. A SUV >2.5–3 is considered to indicate a malignant lesion [89]. Compared to CT imaging alone, higher sensitivities (86–100%) and specificities (69–87%) have been reported for FDG-PET [90], [91] in the detection of head and neck tumors or lymph node metastases. Due to the low spatial resolution, as well as the lack of anatomical landmarks, a precise anatomical mapping is often not possible, especially in the head and neck with its complex anatomy. FDG-PET/CT is a so-called hybrid imaging technique. The functional information of FDG-PET is combined with high spatial resolution CT using image fusion (Figure 23 (Fig. 23)) to allow precise anatomical identification of the site of altered metabolism. Therefore, FDG-PET/CT has become an established imaging technique for the detection of primary tumors and lymph node or distant metastases and also for follow-up and monitoring of the treatment response [89], [92], [93].

Patients should fast before a FDG-PET/CT scan for 6 hours, and a blood glucose level <200 mg/dl immediately before the examination will ensure adequate uptake of FDG into tumor cells. The co-administration of IV insulin may help to reduce the risk of hyperglycemia but may also degrade image quality due to increased FDG storage in muscles and fat tissue. FDG-PET/CT examination of patients with hyperglycemia >200–250 mg/dl should therefore be postponed, if possible, until there is a more favorable metabolic status [85], [93].

A typical whole-body FDG-PET/CT includes the area from the forehead to the mid-thigh. However, there is growing evidence that a dedicated examination protocol for the head and neck region (Figure 23) with a smaller field of view (FoV), thinner slices, and a longer recording time for the individual sections of the PET, so-called *high resolution head and neck PET/CT* (HR HN PET/CT), can improve diagnostic accuracy [93], [94]. The simultaneously acquired CT scan should be contrast enhanced [94].

**Figure 24:**
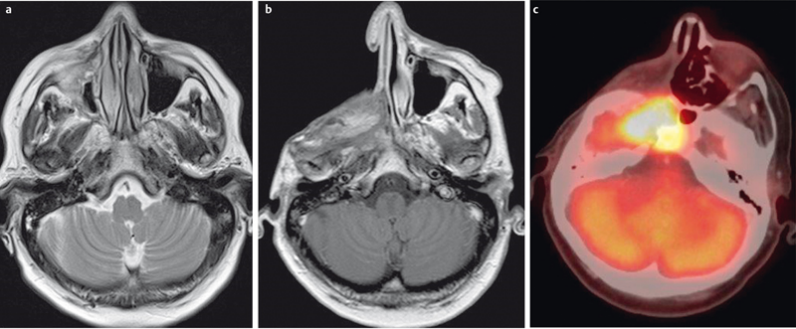
46-year-old female patient with chondrosarcoma of the right maxillary sinus. a) Preoperative axial T2w image demonstrates an inhomogeneous tumor with discrete infiltration of the surrounding soft tissue. b) Contrast-enhanced T1w follow-up image demonstrating extensive scar formation which cannot be differentiated from recurrent tumor. c) FDG-PET/CT performed the next day demonstrating increased metabolism within the scar formation, indicating recurrent disease, which was confirmed by surgery.

FDG-PET/CT examinations are most beneficial for the detection of asymptomatic tumor recurrence (Figure 24 (Fig. 24)) or posttherapeutic residual tumors [95], [96], [97]. In these cases, FDG-PET/CT has a very high sensitivity (90–100%) and a high negative predictive value [93], [98]. This is especially advantageous in patients with significantly altered anatomy after tumor resection and reconstructive surgery [89]. 

Scar formation, especially after extensive surgery, can be difficult to be differentiated from possible residual or recurrent tumor [99]. In a retrospective study of 123 patients by Dunsky et al. [95], FDG-PET/CT identified asymptomatic recurrence in 20% of all patients rated as disease-free by conventional CT. However, this increase in diagnostic yield did not lead to decreased mortality in their patient population. To avoid false positive results, it is crucial to have a distinct time interval between treatment and imaging. For radiochemotherapy, the majority of authors recommend a time interval of 12 weeks [89], [93], [100], [101], [102], and after surgery, the interval before imaging should be at least 4–6 weeks [98], [100]. Because surgical complications, e.g. abscess formation, can lead to false positive findings, interdisciplinary cooperation is necessary to avoid misdiagnosis [93]. FDG-PET/CT is also suitable for monitoring therapeutic response. Chen et al. demonstrated, in a prospective study of 51 patients, that a reduction of SUV <0.64 compared to baseline value is associated with poor prognosis [103]. There is also a correlation between of the prognosis and the preoperative SUV of the tumor or the ipsilateral [104] and contralateral [105] lymph nodes. A tumor SUV >8.5 preoperatively is associated with a significantly increased risk of recurrence [105]. 

**Figure 25:**
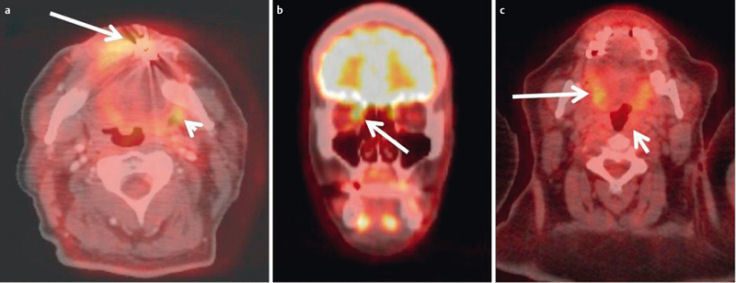
Artifacts and pitfalls in FDG-PET/CT. a) 55-year-old patient with recurrent lip carcinoma. A malignant lesion in the left parapharyngeal space (short arrow) is suggested by misregistration caused by involuntary patient movement. The presumed tumor cannot be assessed in its entirety due to artifacts of metallic dental implants. b) 41-year-old patient with intraorbital metastasis of a nasal squamous cell carcinoma. The tumor cannot be differentiated from the metabolically active medial rectus muscle. c) 64-year-old female patient with CUP syndrome. Histologically proven carcinoma of the left pharyngeal tonsil. The SUV of the tumor was below the SUV of the intrinsic muscle of the tongue due to its low affinity to FDG.

Because FDG is not a tumor-specific tracer, there is a potential risk of misdiagnosis related to artifacts (Figure 25 (Fig. 25)). False positive results may occur due to misregistration of FDG-PET and CT caused by patient movements [85], [89], [93] as well as a low affinity of the tumor to the tracer [93] or a tumor size below the spatial resolution of the PET detector [85]. Especially in the midface and the paranasal sinuses, masking of a tumor by artifacts of metal-containing dental implants or close spatial relation to highly FDG-affine tissue, such as the brain or the extra-ocular eye muscles (Figure 25 (Fig. 25)), may occur [93]. Perineural tumor spread can also be identified by FDG-PET/CT. However, its role for this disease entity in comparison to MR imaging has yet to be defined [106].

## 6 Magnetic resonance imaging (MRI)

**Figure 26:**
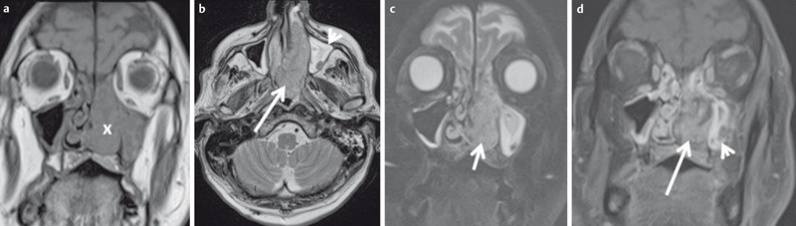
MRI of a 65-year-old patient with histologically proven squamous cell carcinoma of the nasal cavity. a) Coronal T1w image. The tumor (x) is iso-intense to muscle, fat tissue is hyperintense on T1w images. b) Axial T2w image. The tumor is hyperintense to muscle. MRI allows differentiation between hyperintense retention within the maxillary sinus and the tumor. c) Coronal T2w image with fat suppression. With fat tissue appearing hypointense, tumor and inflammatory changes of the maxillary sinus are better delineated. d) Coronal contrast-enhanced T1w images with fat suppression. Contrast enhancement is better delineated with fat suppression. The tumor (arrow) enhances avidly but less compared to normal mucosa (arrowhead).

**Figure 27:**
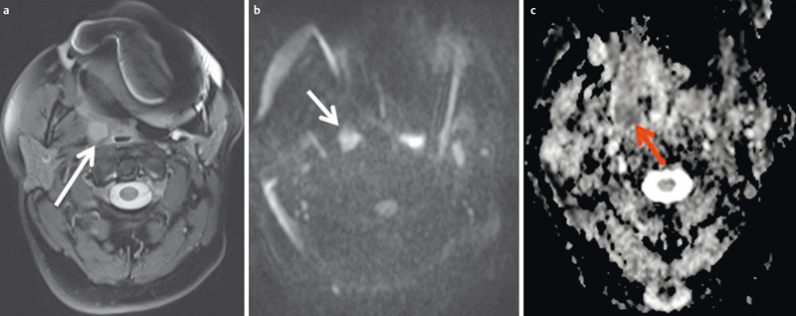
49-year-old patient after resection of a lip carcinoma on the right side. a) Axial T2w images demonstrate a solitary retropharyngeal lymph node (arrow). Significant artifacts due to metallic dental implants. b) The lymph node (arrow) appears hyperintense on diffusion-weighted images. c) The lymph node (arrow) appears hypointense on the ADC map. Calculated ADC was 0.65×10^–3^ mm^2^/s, indicating malignancy. This was confirmed by histology.

MRI relies on the excitation of hydrogen protons in tissues by a high-frequency radiopulse while the patient lies inside a magnetic field. The resulting signals are then registered using a special antenna, the so-called coil. In contrast to CT, tissues are not characterized and differentiated by densities measured in Hounsfield units, but on the basis of their signal intensities in the differently weighted images. A basic distinction is made between T1-weighted (T1w) and T2-weighted (T2w) images (Figure 26 (Fig. 26)). Tissue contrasts can be enhanced by intravenous contrast agents. The standard MR contrast agents are gadolinium-based and induce a T1 signal increase of tissues that accumulate them. With dedicated excitation pulses, the signal from fat, which is hyperintense (= bright) on both T1w and T2w images, can be suppressed (so-called fat saturation). This technique can be used to enhance the conspicuity of edema on T2w images or to emphasize contrast enhancement on T1w images. Diffusion-weighted imaging (DWI) techniques allow the detection and quantification of water diffusion in vivo (Figure 27 (Fig. 27)) and contribute additional information for tissue characterization [107].

The midface should always be imaged with a dedicated head-neck coil [8] while the orbit can be imaged using either a small loop surface coils or the head coil alone or in combination with a surface coil [108]. Surface coils allow a smaller field of view with increased spatial resolution, while the head coil provides a more homogenous signal and allows the simultaneous assessment of the contralateral orbit.

### 6.1 Fractures

Because of their low content of water, cortical bone appear as hypointense (= dark) structures. With exception of the skull base, bony structures of the midface and orbit are too thin to be evaluated by MRI. Therefore MR imaging is the imaging modality of choice in the acute trauma setting [109]. However, T2w images with fat suppression are superior compared to CT for the detection of posttraumatic lesions of the optic nerve [79].

### 6.2 Tumors of the midface

**Figure 28:**
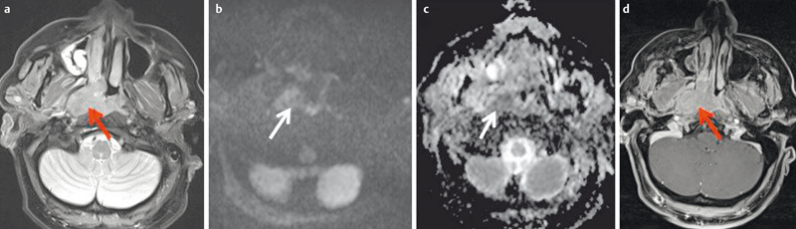
MRI of a 66-year-old patient with a nasal cavity carcinoma extending into the nasopharynx. a) There is marked enhancement on contrast-enhanced T1w image with fat saturation. b) Axial T2w images demonstrate a homogeneous, discretely hypointense tumor (arrow). c) The tumor is hypointense on the ADC-map and the calculated ADC-value is 0.51×10^–3^ mm^2^/s. Histologically confirmed squamous cell carcinoma.

MRI is superior to CT for the differentiation between inflammatory changes and tumor [61]. Inflammatory changes, as well as retained secretions, appear very bright on T2w images due to their high water content [110]. In contrast, due to their high cellularity, most midface tumors appear hypointense on T2w sequences and have an intermediate signal on T1w images [3], [4], [61], [110]. These tumors show significant enhancement after contrast administration (Figure 26 (Fig. 26)). However, there is often a significant overlap between the imaging appearance of benign and malignant tumors [111]. An ADC parameter map (*apparent diffusion coefficient*, ADC) computed from DWI [112] may improve the diagnostic accuracy of MRI. A low ADC (Figure 28 (Fig. 28)) indicates restricted diffusion of water in the tissue, which correlates with tumor cellularity [113]. Therefore, malignant tumors have a significantly lower ADC value (0.87 ± 0.32×10^3^ mm^2^/s) compared to benign tumors (1.35 ± 0.29×10^3^ mm^2^/s) or inflammatory changes (1.50 ± 0.5×10^–3^ mm^2^/s), as demonstrated by Sasaki et al. in a retrospective study of 61 patients [114]. DWI also allows the detection of lymph node metastases before morphological changes become apparent on anatomical MR images. In a prospective study of 301 histologically analyzed lymph nodes, Vandecaveye et al. [115] found 94% sensitivity and 97% specificity for the detection of metastatic lymph nodes <1 cm in diameter using an ADC cut-off value <0.94×10^–3^ mm^2^/s. These findings were confirmed in a prospective study of Barchetti et al. [116] including 239 histologically analyzed lymph nodes. They reported comparable sensitivity and specificity for MR imaging at 3T.

**Figure 29:**
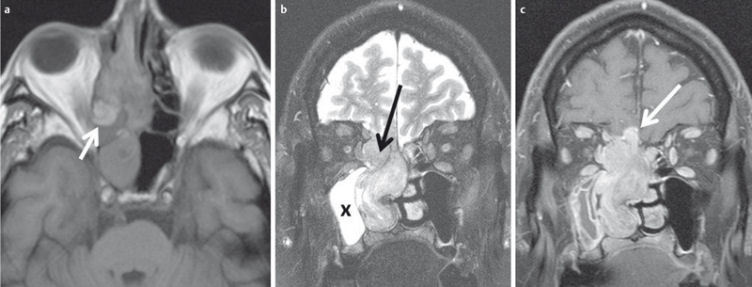
48-year-old patient with histologically confirmed malignant melanoma of the right nasal cavity. a) The tumor appears hyperintense on plain T1w images. b) On T2w images the tumor is inhomogeneously hyperintense and can be clearly differentiated from the hyperintense inflammatory changes of the right maxillary sinus (x). c) The tumor enhances avidly after contrast administration, and there is intracranial tumor growth through the cribriform plate.

In general, MRI does not allow histological classification of lesions [4]. However, a hyperintense tumor on non-enhanced T1w images (Figure 29 (Fig. 29)) makes malignant melanoma the most likely diagnosis. The signal increase in unenhanced T1w images is related to the paramagnetic effects of melanin and is therefore more common in melanotic than in amelanotic tumors [117].

**Figure 30:**
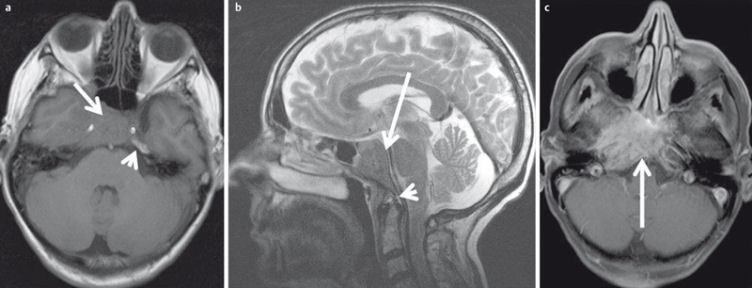
53-year-old patient with squamous cell carcinoma of the nasal cavity and osseous infiltration of the skull base. a) On T1w images the hypointense tumor (arrow) replaces the hyperintense fatty marrow of the skull base (arrowhead). b) On sagittal T2w images (arrow) the tumor also appears hypointense compared to bone marrow (arrowhead). c) There is avid enhancement after contrast administration.

Osseous infiltration of the skull base is better detected by MR imaging than by CT [118]. Particularly, the hyperintense signal of fatty bone marrow should be carefully evaluated to identify areas of low signal intensity as a sign of bone marrow infiltration by tumor on T1w and Tw2 images (Figure 30 (Fig. 30)) [61]. Other common imaging features of osseous infiltration are hyperintense bone marrow signal on T2w images with fat saturation and contrast enhancement [61], which is best appreciated on T1w images with fat saturation.

Intraorbital and intracranial tumor extension can also be better assessed by MR imaging [4]. Linear enhancement of the dura may indicate a reaction to adjacent tumor growth. Nodular enhancement or thickening of the dura >5 mm, on the other hand, indicates dural infiltration with 100% sensitivity and 91% specificity [119] while pial enhancement is the positive proof. Abnormal signal intensities in the extraocular muscles (100%) on T2w images have the highest positive predictive value for orbital infiltration, followed by stranding of orbital fatty tissue (80%) [120], [121], [122].

**Figure 31:**
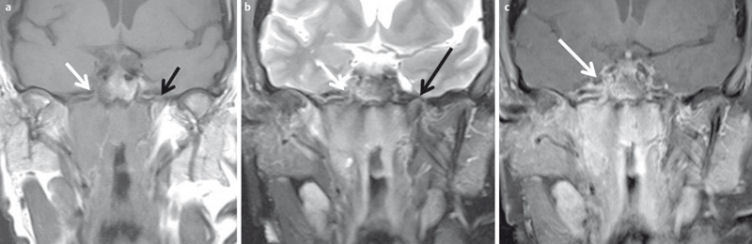
MRI criteria of perineural tumor spread. 62-year-old patient with histologically proven carcinoma of the anterior ethmoid cells and new onset of facial pain on the right side. a) Coronal T1w images with loss of fat signal (white arrow) of the oval foramen in comparison to contralateral side (black arrow). b) Hyperintense signal on T2w images with fat suppression of the oval foramen (arrow) in comparison to contralateral side (dotted arrow). c) Avid enhancement within the oval foramen on the right side (arrow), which is widened compared to the contralateral side.

A new onset of cranial nerve palsy in patients with known malignancy of the midface is highly suspicious of perineural tumor *spread* (PNS). Neurological symptoms rarely occur before detection of the primary tumor. MRI is the imaging modality of choice for detecting of PNS. Typical imaging findings (Figure 31 (Fig. 31)) are loss of fat signal in the foramina of the skull base or the pterygopalatine fossa, enhancement of the affected nerve after contrast administration, and, in advanced stages, widening of the foramina or atrophy of the muscles of mastication due to denervation [123].

Early scar formation and recurrence or residual tumor may exhibit similar contrast enhancement and can therefore pose a diagnostic dilemma for conventional MRI. DWI may overcome this limitation. In a prospective study of 32 patients with known recurrent tumor, Abdel Razek et al. [107] showed that an ADC <1.3×10^–3^ mm^2^/s indicates recurrent disease with 84% sensitivity and 90% specificity and a positive predictive value of 94%. 

DWI may also predict the clinical course. Lambrecht et al. [124] postulate that pretherapeutic ADC is an independent prognostic factor for survival of patients with midface tumors. They prospectively examined 161 patients with a median follow-up period of 50 months. Similar results were found by Nakajo et al. [125] in their prospective study of 26 patients. An ADC value <0.88×10^–3^ mm^2^/s significantly correlates with an unfavorable clinical outcome. The predictive value is comparable to that of a markedly increased SUV in FDG-PET/CT at baseline. 

### 6.3 Orbit

**Figure 32:**
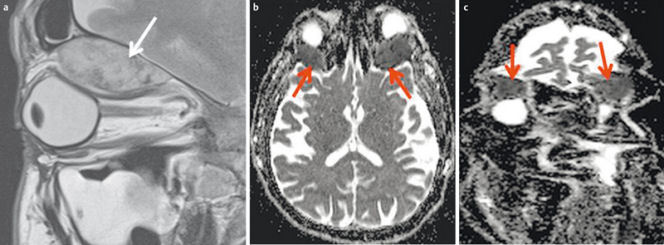
44-year-old patient with new onset of diplopia and progressive loss of vision in both eyes. a) T2w images demonstrate a subperiostal inhomogeneous lesion with smooth margins. b) Axial ADC map demonstrates bilateral lesions (arrow). ADC-value is >1.0×10^–3^ mm^2^/s on both sides, indicating a benign lesion (histologically proven hematoma due to coagulopathy). c) Coronal ADC map with significant distortion artifacts due to air-tissue interfaces and involuntary eye movement.

**Figure 33:**
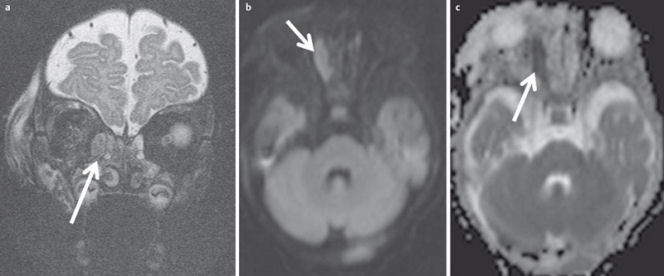
4-month-old child with new orbital swelling and fever after sinusitis. a) Coronal T2w images with fat saturation demonstrate a small hyperintense lesion at the medial aspect of the orbital wall. There are no signs of orbital cellulitis. b) The lesion appears hyperintense on diffusion-weighted images. c) and hypointense on the ADC-map, indicating abscess formation. This was confirmed by surgery.

With its excellent soft tissue contrast, MRI is the modality of choice for imaging the orbit [77]. Benign and malignant orbital lesions also have a significant overlap of morphological criteria. DWI may improve the differentiation of benign and malignant orbital lesions. Based on a prospective study of 47 patients, Sepahdari et al. [126] defined an ADC value <1.0×10^–3^ mm^2^/s as a cut-off for the differentiation between benign and malignant lesions with 64% sensitivity and 84% specificity. In particular, lesions which appear hypointense in T2-weighted images (Figure 32 (Fig. 32)) can be better differentiated. Fatima et al. identified a different cut-off of 0.84×10^–3^ mm^2^/s in their prospective study of 39 patients. With this corrected threshold, they differentiated benign and malignant lesions with 83% sensitivity and 86% specificity [127]. DWI is also suitable to distinguish orbital cellulitis from abscess formation (Figure 33 (Fig. 33)) at an early stage [128]. In patients with an endocrine orbitopathy, the ADC value can be used as a quantifiable biomarker for assessing disease activity, as Politi et al. demonstrated in a prospective study [129]. 

A major limitation of orbital DWI is the vulnerability to artifacts [130], either caused by eye movement or at the interface to the aerated paranasal sinuses (Figure 32 (Fig. 32)). This limitation can be overcome by using optimized sequences, allowing reliable ADC quantification of lesions in the orbital space [131]. 

MRI also allows perfusion weighted imaging. This can be performed as T2w Dynamic susceptibility contrast enhanced (DSCE) imaging, which is comparable to PCT, and as T1w contrast enhanced imaging [132]. A recent meta-analysis by Bernstein et al. demonstrated a good correlation between MR-derived perfusion parameters and clinical outcome [133]. However, PWI of the midface and orbit is also limited by severe artifacts. Therefore, it is not an established imaging technique in clinical routine [133].

## 7 FDG-PET/Magnetic resonance imaging (FDG-PET/MRI)

**Figure 34:**
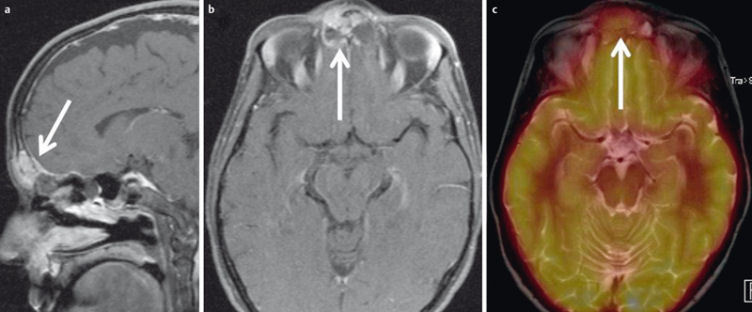
FDG-PET/MRI of a 47-year-old female patient with a 3-month history of pressure sensation in the frontal head. a) Sagittal contrast-enhanced T1w images showing an enhancing tumor within the frontal sinus and linear enhancement of the mildly thickened dura (arrow). b) The posterior and anterior cortical bone of the frontal sinus is thinned by the tumor and there is intracranial tumor growth (arrow). c) FDG-PET/MRI demonstrates significantly increased metabolism. Histologically proven Ewing sarcoma.

The wider availability of FDG-PET/MR systems (Figure 34 (Fig. 34)) has increased the use of this modality for the diagnostic assessment of head and neck tumors [134]. Theoretical advantages over FDG-PET/CT are the better soft-tissue contrast of MRI [135] and the option of combining PET information with functional MRI techniques, such as DWI [136]. Especially in the midface, FDG-PET/MRI has the added advantage of being less susceptible to artifacts caused by metallic dental implants [137]. Partovi et al. studied 14 patients who had undergone FDG-PET/CT and FDG-PET/MRI and found no significant difference in terms of diagnostic accuracy [138]. In another study, fusion of DWI data with FDG-PET data was found to have no impact on diagnostic yield [139]. In a study of lymph node metastases in 12 patients, FDG-PET/MRI had 80% sensitivity versus 70% for MRI alone [140].

Although the availability of these hybrid systems is increasing, their number is still small. Therefore, the role of FDG-PET/MRI in the diagnostic evaluation of head and neck tumors remains to be defined in further studies [135].

## Summary

Conventional radiography has been replaced by computed tomography (CT) and magnetic resonance imaging (MRI) in the diagnostic evaluation of the midface and orbit.

Low-dose CT is the imaging modality of choice in patients with chronic rhinosinusitis. Alternatively, digital volume tomography can be used. However, scientific evidence is still sparse, and no specific recommendations for its use have been made by the major societies.

For all other conditions, axial CT datasets should be acquired with secondary reconstruction in axial and coronal planes in the soft tissue and bone window-level setting. Use of an IV contrast agent is required for assessing inflammatory and neoplastic conditions. The role of perfusion CT in the routine clinical setting remains to be defined.

Eye protection should be used to decrease radiation exposure to the lens.

FDG-PET/CT is the imaging modality of choice for patients with recurrent disease or for patients with cancer of unknown primary.

MRI is the modality of choice for imaging the orbit, except in the acute trauma setting and for the detection of intracranial complications of inflammatory or neoplastic conditions of the midface.

Due to limited availability, the role of FDG-PET/MRI in head and neck imaging has to be further evaluated.

With the use of all imaging modalities, interdisciplinary cooperation is the key to avoiding unnecessary examinations and misdiagnosis.

## Competing interests

The author declares that he has no competing interests.
